# Cerebral Autosomal Dominant Arteriopathy With Subcortical Infarcts and Leukoencephalopathy (CADASIL) Presenting as Migraine

**DOI:** 10.7759/cureus.15355

**Published:** 2021-05-31

**Authors:** Muhammad Atif Ameer, Beenish Sohail Bhutta, Neelma Asghar, Muhammad Talha Haseeb, Raya Nasir Abbasi

**Affiliations:** 1 Department of Internal Medicine, Punjab Rangers Teaching Hospital, Lahore, PAK; 2 Department of Internal Medicine, Shaikh Zayed Hospital, Lahore, PAK

**Keywords:** cadasil, subcortical ischemic leukoencephalopathy (cadasil), stroke, migraine disorder, genetic

## Abstract

Cerebral autosomal dominant arteriopathy with subcortical infarcts and leukoencephalopathy (CADASIL) is a hereditary arteriopathy with a genetic predilection for the cerebral vessels. It is caused by mutations in the NOTCH3 gene and commonly occurs in middle-aged individuals. Clinical manifestations range from stroke, transient ischemic attack, and migraine to neuropsychiatric symptoms. We present a case of a 40-year-old patient who came in with headache, blurry vision, progressive right-sided weakness, and behavioral changes. The diagnostic workup included several possibilities, including central nervous system (CNS) infection, stroke, transient ischemic attack, and inherited disorders like mitochondrial encephalopathy, lactic acidosis, and stroke-like episode (MELAS). After proper systemic and genetic workup, we diagnosed this as a case of CADASIL.

## Introduction

Cerebral autosomal dominant arteriopathy with subcortical infarcts and leukoencephalopathy (CADASIL) is a hereditary arteriopathy caused by a genetic mutation in the NOTCH3 gene. It commonly manifests in middle-aged individuals and may present initially with a wide spectrum of symptoms, including migraines with aura, mood disturbances, cognitive impairment, or dementia [1]. The prevalence of this disease is variable in the literature due to the rarity of the condition. However, a Scottish study reported its prevalence to be 1.98 in 100,000 adults with a mutation frequency of 4.14 in 100,000 patients [2].

Mutation of the NOTCH3 gene at chromosome 19q12 is the hallmark of CADASIL. A series of NOTCH3 mutations can cause protein misfolding and receptor aggregation, which accumulates within small vessels and obstructs blood flow, and is responsible for recurrent ischemic subcortical infarction [3]. Migraine with aura is the first and most frequent symptom to appear in around 20-40% of the affected patients. Sub-cortical ischemic events usually range from a pure motor/sensory to ataxic hemiparesis occurs in 60-85%; mood disturbances/apathy in 20%; cognitive impairment in 10%; and memory loss and seizures in 5-10% of the patients [1].

## Case presentation

A 40-year-old right-handed male patient with a past medical history of migraine presented in the emergency department of a tertiary care hospital with complaints of headache, blurry vision, a progressive right-sided weakness for the past seven hours. He was diagnosed with migraine with aura for two years and had similar flares in the past but did not seek medical help as symptoms always resolved within a few hours with no residual deficits. His blood pressure at the time of presentation was 175/95 mmHg in the right arm; oral temperature 100 F; pulse 89 bpm. Headache was sudden without aura, 8/10 in intensity, unilateral on the right side, pulsatile, associated with vomiting, aggravated by speaking and light, and relieved by dimming the lights. Physical examination showed decreased power (0/5) and increased tone on the right side of the arm. The rest of the motor examination showed normal bulk, tone, and power. Neck extension and flexion were 5/5. Cranial nerves III-XII were intact. Babinski’s sign was positive on the right side. The rest of the physical examination was unremarkable. The patient was started on dihydroergotamine (DHE) and nitroglycerin in the emergency department. Non-contrast computed tomography (CT) was negative for acute bleeding. During the emergency department stay, the patient had an episode of generalized seizure, which lasted for four minutes and was managed by IV lorazepam.

**Figure 1:**
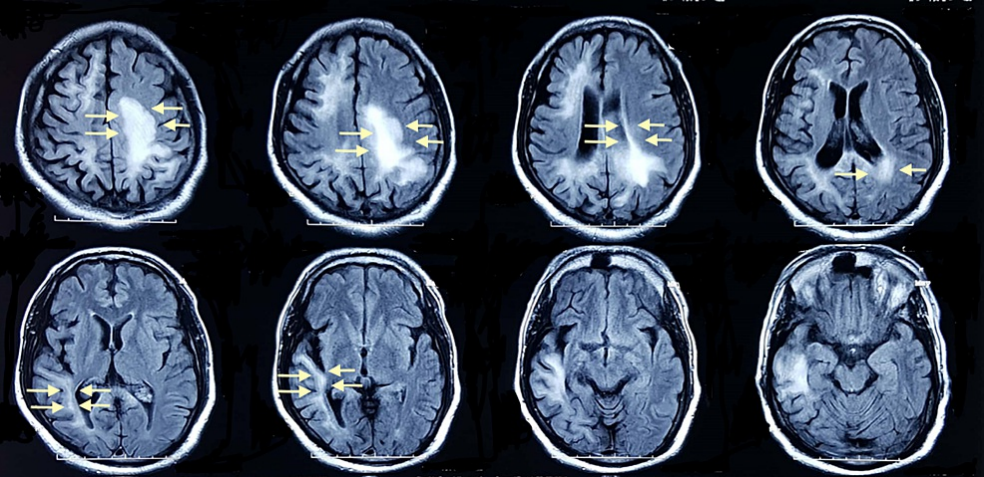
Flair showing hyperintensities in the occipital lobe, parietal lobe, and periventricular region.

The patient was admitted to the medical floor. Magnetic resonance imaging (MRI) was ordered, which showed a restricted area of diffusion at Flair in the occipital, parietal, and periventricular region (Figure 1).

The carotid duplex showed no hemodynamically significant stenosis bilaterally. Trans-esophageal echocardiography was also unremarkable. The differentials included central nervous system (CNS) infections, transient ischemic attack (TIA), acute disseminated encephalomyelopathy (ADEM), multiple sclerosis (MS), space-occupying lesion (SOL), CNS vasculitis, inherited disorders like mitochondrial encephalopathy, lactic acidosis, and stroke-like episode (MELAS), and CADASIL.

Fundoscopy was not performed due to photophobia. Lumbar puncture (LP) showed normal opening pressure, cell count, glucose, protein, and no oligoclonal bands. Lyme disease serology and Venereal Disease Research Laboratory test were negative. Erythrocyte sedimentation rate (ESR) and C-reactive protein (CRP) were slightly raised. Anti-myelin oligodendrocyte glycoprotein (anti-MOG) was tested and came back positive. The patient was started on steroid therapy, donepezil for cognitive decline, furosemide, and captopril for blood pressure control and referred to the rehabilitation center to manage the physical deficit. Meanwhile, a skin biopsy was done for genetic testing, which showed a missense mutation in NOTCH3 at exon 19, confirming the diagnosis. After four weeks, the patient showed improvement in the right-sided weakness with well-controlled blood pressure. Steroids were discontinued at this moment after tapering for one week.

## Discussion

To our knowledge, the first case of CADASIL was documented in 1955. From the 1950s to the late 1980s, various cases of vascular dementia with Binswanger's like arteriopathy were described [4]. CADASIL is the most common hereditary stroke disorder of all the rare diseases affecting the brain's white matter. The key difference from other vascular disorders is the accumulation of osmiophilic granular material in brain vasculature. In 1996, the first study that mapped out the genetic mutations of the NOTCH3 gene was published [5]. Since then, more than 200 mutations have been reported. NOTCH3 is a transmembrane receptor most commonly present in smooth muscle cells and pericytes in local blood vessels. Inactivation of cysteine residue results in receptor misfolding and aggregation [6]. This NOTCH3 domain within the vessels results in the formation of granular osmiophilic material, which has a critical diagnostic role in CADASIL. The receptor has a large extracellular domain with 34 epidermal growth factor-like repeats encoded by exons 2-24, where the repeats codons are most frequently present [7].

Clinical presentation ranges from as simple as a headache to as complex as a weakness of middle-aged people's limbs. Migraine with aura is the most common presenting complaint [8]. It is usually associated with visual and sensory symptoms. However, in some patients, motor and brainstem symptoms are also present. Migraine can also occur without aura. Other manifestation includes weakness or paralysis of the face, arm, leg, foot, or toes; sudden numbness; difficulty walking; difficulty speaking; clumsiness of a hand or arm; weakness or paralysis of ocular muscles; cognitive decline primarily related to executive function; verbal/visual memory, reasoning, and language deficit; seizures; and emotional disturbances like apathy. Although rare, some patients report spinal symptoms, depending on the type of vasculature involved [9]. A similar condition known as cerebral autosomal recessive arteriopathy with subcortical infarcts and leukoencephalopathy (CARASIL) has a similar presentation. However, the critical difference is the absence of migraine with aura with cognitive decline occurring before other symptoms [10].

The diagnosis of CADASIL is usually made from radiological (MRI) and clinical correlation. However, genetic testing is required for some of the cases. By the age of 35, most patients have abnormal magnetic resonance imaging (MRI) findings. Computerized tomography (CT) scans usually miss the early finding but should be done to exclude acute bleeding. MRI during the early stages of the disease can show non-specific periventricular and subcortical hyperintensities [11]. However, it can also occur in uncontrolled hypertension, so symptoms will play an important role in diagnosing CADASIL [12]. MRI scan of MELAS is very similar to CADASIL. However, MELAS will present early (infancy or childhood) with myopathy symptoms, hearing loss, and visual loss, and imaging will reveal vasogenic edema. The mass effect is also peculiar to MELAS. Other diagnostic modalities include fluorodeoxyglucose positron emission tomography (FDG-PET) and diffusion tensor imaging (DTI) [1]. Recent studies like optical coherence tomography angiography (OCT-A) is a recent tool for ophthalmic imaging is a very effective and non-invasive technique to diagnose early signs in CADASIL patients [11].

Chabriet et al. conducted a study using a standardized questionnaire to see the migraine symptoms in 378 patients of CADASIL. 54.5% of the patients reported a history of migraines. The majority of the patients (84%) had a history of migraine with aural symptoms, with a female predominance (62.4%) [13]. Numerous other studies identified migraine as the most commonly encountered symptom in patients of CADASIL. The vast majority of the patients use simple analgesics and beta-blockers as the abortive therapies for such migraines [13-14]. 

Currently, there is no treatment of proven efficacy [3], and treatment mostly includes symptomatic relief and minimizing risk factors, particularly maintaining blood pressure to close range. A multi-disciplinary approach, including neurologists, internists, rehabilitation specialists, and psychiatrists, is essential to prevent the long-term implications of the disease. Migraine should be treated depending on the occurrence, either acutely or prophylactically with nonsteroidal anti-inflammatory drugs (NSAIDs), triptans, dihydroergotamine (DHE), opioids, beta-blockers, calcium channel blockers, or tricyclic anti-depressants (TCA) [15]. Minimizing risk factors like control of blood pressure, cholesterol, diabetes mellitus (DM), and quitting smoking also plays an important role. Thrombolytic therapy should be avoided to minimize intracranial bleeding risk and should only be started in particular cases [3]. Patients with CADASIL tend to have reduced life expectancy that depends on several factors, including genotype/phenotype co-relation, gender, hypertension, and diabetes. Pneumonia has been reported as the most common cause of death in diagnosed patients [12]. Routine follow-up with internists, neurologists, and psychiatrists should be encouraged to maximize the patients' functionality.

## Conclusions

The diagnosis of a rare disease is always challenging in a medical setup. Listing CADASIL, along with other causes of motor weaknesses and atypical mood changes, would help the clinician to diagnose this rare condition. Although genetic testing is not essential for the diagnosis of CADASIL, it should also be done in suspected cases, including stroke, at an early age without significant co-morbidities. The family members of the patients should be encouraged to undergo genetic testing for a timely and prompt diagnosis and treatment. Rehabilitation is the main focus of treating cognitive disabilities in addition to symptomatic treatment. Early diagnosis of CADASIL can lead to better management of the risk factors, consequently improving the quality of life.
